# Prevalence, type, and reasons for missed nursing care in municipality health care in Sweden – A cross sectional study

**DOI:** 10.1186/s12912-022-00874-6

**Published:** 2022-04-24

**Authors:** Ingrid Andersson, Anna Josse Eklund, Jan Nilsson, Carina Bååth

**Affiliations:** 1 Department of Health Sciences, Faculty of Health, Science, and Technology, Karlstad University, 651 88 Karlstad, Sweden; 2 Faculty of Social and Health Sciences, Inland Norway University of Applied Sciences, N-2418 Elverum, Norway; 3 Faculty of Health, Welfare, and Organisation, Østfold University College, NO-1757 Halden, Norway

**Keywords:** aged, BERNCA-NH, elderly people, home care services, missed nursing care, nursing homes, patient safety, Prevalence

## Abstract

**Background:**

With an ageing population, there is an increasing need for care, both as home care and in nursing homes. However, some needed care is not carried out for different reasons, which can affect patient safety. The aim of the study was to describe prevalence, type, and reasons for missed nursing care in home care and nursing homes, from nurses’ perspective.

**Methods:**

A cross sectional design with quantitative and qualitative approach. A Swedish version of Basel Extent of Rationing of Nursing Care for nursing homes and 15 study specific questions were answered by 624 registered nurses, enrolled nurses, or nurse assistants. Both descriptive and analytical, independent-samples t-test, analyses were used. Qualitative content analysis was used for the open-ended question.

**Results:**

The care activity most often missed in home care was: ‘set up or update care plans’ (41.8%), and in nursing homes: ‘scheduled group activity’ (22.8%). Reasons for missed nursing care were lack of preparedness for unexpected situations, obstacles in a deficient work environment, unsatisfactory planning in the organisation, and/or shortcomings related to the individual.

**Conclusion:**

Not all care activities needed are performed, due to reasons such as lack of time or organisational issues. Missed nursing care can lead to adverse events and affect patient safety. It is important to be aware of missed nursing care and the reasons for it, which gives a possibility to initiate quality improvement work to ensure patient safety.

## Introduction

An ageing population is a challenge for health care systems in meeting the growing care needs [1]. At the same time, there is a general shortage of health workers as well as a shortage of adequately educated health workers [2].

The phenomenon missed nursing care can be described using different concepts, referring to nursing care within the areas of nurse´s responsibility [3, 4]. It is a description that includes all nursing care that should have been carried out but was omitted [5, 6], fully or partly [6], or delayed [5, 6]. It can also be seen as an indication of omission [5, 6], where the necessary nursing care is performed incompletely [6–10]. There is no consensus in what concept to use [3]. Henceforth in this paper, the concept ‘missed nursing care’ will be used.

Reasons for missed nursing care can be related to lack of staff, unexpected increase in the number of patients or the required needs [11], or inadequate staffing in relation to needed competences [12, 13], which in turn can be an explanation for how nurses make decisions and prioritize care [14]. Prioritization of nursing care can be dilemmas where staff have to make difficult decisions, sometimes based on assessments being made to determine what care is the most important [15]. Missed nursing care can also be seen as a contributing factor to adverse events and can affect patient safety [4], therefore it should be of interest to nurses and management.

### The Swedish municipal context 

In Swedish municipalities, people in working age are decreasing, at the same time the number of older people living at home in need of care is increasing, as is the complexity of their care needs. [16]. This has led to an increased demand of advanced nursing care in home care and nursing homes [17]. Sweden consists of 290 municipalities divided into 21 regions [18]. According to the Swedish Health and Medical Services Act [19], municipalities should offer health services to residents. The Social Services Act ascribes an obligation to meet individuals' needs for support and care, either as home care or in nursing homes [20]. In Swedish municipalities, registered nurses are responsible for the care given to older people, however the majority of care is carried out by enrolled nurses or nurse assistants, with or without formal competence [21]. The enrolled nurses or nurse assistants can also perform interventions after receiving a delegation from a registered nurse [22].

A primary goal for all health care workers are to provide care of high quality for all people on equal terms. Despite this, the phenomenon missed nursing care exists. This increasing demand for older people to need care, as home care or in nursing homes, can be seen both internationally and nationally. However, research on missed nursing care in Swedish municipal health care for older people is lacking, despite the fact that studying missed nursing care is one way to identify areas in need of improvement in order to ensure patient safety and quality of care.

## Methods

### Aim

The aim of the study was to describe prevalence, type, and reasons for missed nursing care in home care and nursing homes, from nurses’ perspective.

### Design

A cross-sectional design was used.

### Setting and sample

The study was conducted in both home care and nursing homes in eight medium-sized municipalities with urban and rural area, in one region in Sweden. The inclusion criteria to participate were: health care staff working with older people, as registered nurses, enrolled nurses or nurse assistants (hereafter referred to as nurses) with or without formal education, who were either permanently or temporarily employed. Exclusion criteria were: nurses on an extended period of leave, e.g. parental leave, sick leave, and nurses who do not work with direct care. A total of 3293 were invited to participate, and 671 responded. Of these, 93.0% (*n* = 624) stated their workplace as home care or in nursing homes.

### Questionnaire

The questionnaire consisted of five parts with a total of 64 items and two open-ended questions. In the present study, two parts concerning missed nursing care were included; the 20 items questionnaire Basel Extent of Rationing of Nursing Care for nursing homes (BERNCA-NH), Swedish version, and 15 study specific items. One open–ended question was included, and five demographic questions about gender, age, education, and employment.

### Basel Extent of Rationing of Nursing Care for nursing homes

The questionnaire BERNCA-NH, (19 items), was developed and validated by Zúñiga, Schubert, Hamers, Simon, Schwendimann, Engberg and Ausserhofer [23]. The instrument was translated into a Swedish version, containing 20 items, as one item that contained two activities was separated, (Cronbach´s alpha, 0.91). The starting point for the questionnaire was care that was *‘necessary and usual but could not be performed or partly performed because of lack of time or high workload’*. The items should be answered from the condition; *How often in your last seven working days did it happen that…* after that the items were listed as activities that have not been carried out. The answer options were: *never, seldom, sometimes,* and *often,* where 'never' stands for never missed nursing care and so on to ‘often’ missed nursing care. Participants were also given the possibility to answer: *activity not necessary* or *not within my responsibility*.

### Study specific items

The questionnaire included 15 additional study specific items about missed nursing care. These items complemented BERNCA-NH, with additional items related to common nursing activities in municipal health care for older people, e.g. ‘serving food while it is still hot’, ‘acting if abuse occurred’. The items had the same structure as the questionnaire BERNCA-NH and were answered in the same manner. One open-ended question: *What can you see as reasons for missed nursing care?,* was added to describe the nurses´ perceptions in their own words.

### Data collection.

Data were collected from October 2019 to January 2020. Contact was first made with the manager of the community health care for older people in eight municipalities in Sweden, to obtain permission to conduct the study, and to get access to the nurses e-mail addresses at work or home addresses. The distribution of the questionnaire was done, upon an agreement with the manager (since all nurses did not have work e-mail addresses). Either it was sent as a link in an e-mail or delivered as a hard copy. The hard copy was either delivered at staff meetings for all nurses or to the nurses’ home addresses. Two reminders were sent out.

### Data analysis

Collected data were analysed using descriptive statistics with percent, mean (M), and standard deviation (SD). The analytical statistics independent-samples t-test was used to identify significant differences between groups, (home care versus nursing homes), using a significant value of* p* ≤ 0.05 [24]. All statistical analyses were made using IBM SPSS Statistics 27. The analyses were only done on the four options *never, seldom, sometimes,* and *often*. Missed answers for the different items were low and ranged from 1.5 to 2.7%.

The open-ended question was answered in 192 written comments that were either single-word or full sentences. For analysis, an inductive approach was used, searching for similarities and differences [25]. Following Graneheim and Lundman [26] qualitative content analysis, first the sentence units were found and condensed. After that, the sentence units were abstracted in the creation of codes, and later on described as categories based on different content areas.

### Ethical considerations

The participants received written information about the study, participation was voluntary and anonymous, and sending in the questionnaire, by mail/e-mail, was considered as informed consent. The study was approved by the Swedish Ethical Review Authority (Dnr: 2019–04,109) and followed the ethical standards as described in The Declaration of Helsinki [27].

## Results

A total of 624 nurses working in home care (*n* = 265) or in nursing homes (*n* = 359) participated in the study. Most of the participants worked as enrolled nurses, and the majority of participants had been working for more than five years. Participating nurses were aged between 19 and 67 years, with a mean age of 48.1 year (SD 12.0). For detailed demographic information, see Table 1.

**Table 1:** Demographic of the nurses

	**Total**		**Home care**		**Nursing home**	
	*n*	%	*n*	%	*n*	%
*Profession*						
Registered nurse	42	6.8	23	8.8	19	5.3
Enrolled nurse	510	82.3	202	77.1	308	86.0
Assistant nurse	68	11.0	37	14.1	31	8.7
*Gender*						
Female	587	94.4	242	91.7	345	96.4
Male	34	5.5	21	8.0	13	3.6
Other	1	0.2	1	0.4	0	
*Age*						
19–29 year	57	9.6	33	12.7	24	7.1
30–39 year	101	16.9	53	20.5	48	14.2
40–49 year	119	20.0	50	19.3	69	20.5
50–59 year	204	34.2	78	30.1	126	37.4
60–67 year	115	19.3	45	17.4	70	20.8
*Total of years in profession in municipality*						
< 1 year	14	2.3	11	4.3	3	0.9
1 – 2 years	30	4.9	17	6.6	13	3.7
> 2 – 5 years	84	13.8	47	18.3	37	10.6
> 5 years	479	78.9	182	70.8	297	84.9

### Prevalence and types of missed nursing care

The nurses working in home care reported seldom missed care activities ranging from 4.3% to 28.6%, sometimes missed nursing care ranging from 0.4% to 30.0%, and often missed nursing care ranging from 0.0% up to 41.8%. Ten of 35 care activities were report as missed to some extent (seldom, sometimes, often) half of the times. In home care the most often reported care activities to be missed were: ‘set up or update care plans’ (41.8%), and ‘necessary conversation with family’ (30.0%). Fourteen care activities were reported as never missed in 70% or more of the time. The least missed care activities were not ‘acting if abuse occurred’ (94.5%), and ‘doing a nursing task when not having a delegation’ (93.5%), see Table 2 and 3.

**Table 2:** BERNCA-NH, comparison of missed nursing care between nurses in home care versus nursing homes

**BERNCA-NH**	**Home care (= 265)**							**Nursing home (= 359)**							
*Missed nursing care activities*	**Valid responses**	**Mean**	**Standard** **Deviation**	**Never†** **%**	**Seldom** **%**	**Some-times** **%**	**Often** **%**	**Valid responses**	**Mean**	**Standard** **Deviation**	**Never†** **%**	**Seldom** **%**	**Some-times** **%**	**Often** **%**	**-value‡**
**Sponge bath/partial sponge bath/skin care (total)**	241	1.79	0.97	52.3	22.8	18.3	6.6	328	1.70	0.92	56.1	22.6	16.2	5.2	*ns*
**Oral hygiene**	227	2.07	1.09	41.9	22.9	21.1	14.1	316	2.00	1.03	43.7	22.2	25.0	9.2	*ns*
**Assist food intake**	208	1.55	0.85	63.9	21.2	10.6	4.3	307	1.50	0.79	65.5	22.8	8.5	3.3	*ns*
**Assist drinking**	221	1.39	0.67	70.1	21.3	7.7	0.9	322	1.46	0.79	68.9	19.3	8.4	3.4	*ns*
**Mobilisation/change of position**	233	1.37	0.65	70.8	21.9	6.4	0.9	330	1.49	0.78	66.4	20.3	11.2	2.1	*ns*
**Leave a care recipient in urine/stool longer than 30 min**	244	1.45	0.78	70.1	18.0	9.0	2.9	327	1.54	0.79	62.7	23.5	11.3	2.4	*ns*
**Emotional support**	257	1.90	1.02	47.1	26.8	15.2	10.9	345	2.12	1.00	33.6	32.2	22.9	11.3	0.009**
**Necessary conversation with care recipient**	261	2.16	1.12	36.8	28.4	16.5	18.4	347	2.37	1.03	25.1	29.1	30.3	15.9	0.022*
**Necessary conversation with family**	232	1.70	0.88	54.7	23.7	18.5	30.0	303	1.66	0.80	53.1	29.7	15.5	1.7	*ns*
**Toileting/continence training**	236	1.77	0.96	53.0	24.2	15.7	7.2	321	1.97	0.95	37.4	37.1	16.8	8.7	0.016*
**Allow necessary time for care recipient to perform care themselves**	231	1.87	1.03	48.9	27.3	12.1	11.7	316	1.89	0.93	42.1	34.2	16.5	7.3	*ns*
**Monitoring care recipient as care worker felt necessary**	226	1.66	0.91	58.8	21.7	14.2	5.3	330	2.14	1.06	36.7	26.1	23.9	13.3	0.000**
**Monitoring of confuse/cognitively impaired care recipients and use of restraints/sedatives**	227	1.67	0.87	56.4	24.7	15.0	4.0	326	2.21	1.04	32.2	27.3	27.6	12.9	0.000**
**Keep care recipients waiting who rung**	227	2.02	1.00	39.6	27.3	24.2	8.8	334	2.10	0.95	31.1	36.2	23.7	9.0	*ns*
**Studying care plans at the beginning of shift**	258	2.48	1.12	25.6	25.2	24.4	24.8	352	2.18	1.02	32.1	30.1	25.6	12.2	0.001**
**Set up or update care recipients care plans**	233	2.47	1.11	26.6	21.5	30.0	41.8	324	2.28	1.05	29.3	28.4	27.2	15.1	0.038*
**Documentation of care**	262	2.27	1.06	30.2	28.6	25.6	15.6	348	2.08	0.99	35.9	29.6	25.0	9.5	0.027*
**Scheduled single activity with a care recipients**	217	1.91	1.03	47.5	24.9	17.1	10.6	279	2.54	1.04	21.5	22.6	36.2	19.7	0.000**
**Scheduled group activity with several care recipients**	118	1.97	1.12	47.5	25.4	10.2	16.9	241	2.48	1.12	26.6	21.6	29.0	22.8	0.000**
**Cultural activity for care recipients**	127	1.67	0.94	56.7	28.3	6.3	8.7	250	1.87	0.91	42.8	33.2	18.0	6.0	0.044*

^†^range 1 “never” to 4 “often”, lower mean indicates less missed nursing care.^‡^ Independent-samples t-test *significance ≤ 0.05, **significance ≤ 0.01

**Table 3:** Study specific items, comparison of missed nursing care between nurses in home care versus nursing homes

**Study specific items**	**Home care (= 265)**							**Nursing home (= 359)**							
*Missed nursing care activities*	**Valid responses**	**Mean**	**Standard** **Deviation**	**Never†** **%**	**Seldom** **%**	**Sometimes** **%**	**Often** **%**	**Valid responses**	**Mean**	**Standard** **Deviation**	**Never†** **%**	**Seldom** **%**	**Sometimes** **%**	**Often** **%**	**-value‡**
**Giving prescribed medication**	255	1.12	0.37	89.4	9.8	0.4	0.4	342	1.17	0.51	88.0	7.3	4.1	0.6	*ns*
**Giving prescribed medication within 30 min**	236	1.33	0.62	74.6	18.6	5.9	0.8	332	1.30	0.61	76.5	18.1	4.2	1.2	*ns*
**Serving food still hot**	220	1.20	0.49	84.1	12.7	2.7	0.5	302	1.33	0.64	75.5	16.9	6.6	1.0	0.007**
**Assistance while food still hot**	200	1.23	0.56	83.0	11.5	5.0	0.5	295	1.53	0.75	61.7	25.4	11.5	1.4	0.000**
**Assessing care needs**	229	1.38	0.62	69.0	24.5	6.1	0.4	324	1.38	0.62	68.2	25.9	5.2	0.6	*ns*
**Care plan meeting with other professions**	174	1.56	0.93	66.1	19.5	6.3	8.0	267	1.52	0.77	62.2	25.8	9.4	2.6	*ns*
**Reporting on to staff**	258	1.34	0.61	72.9	20.5	6.2	0.4	349	1.30	0.58	75.1	20.6	3.4	0.9	*ns*
**Advocacy for the elderly**	219	1.26	0.55	78.5	16.9	4.1	0.5	317	1.29	0.58	77.3	17.4	4.7	0.6	*ns*
**Acting if abuse occurred**	182	1.06	0.26	94.5	4.9	0.5	0.0	264	1.16	0.50	89.0	7.6	2.3	1.1	0.009**
**Supervising student**	158	1.42	0.74	70.3	19.6	7.6	2.5	213	1.52	0.80	64.8	20.7	12.2	2.3	*ns*
**Participating in education/courses**	195	1.23	0.53	81.5	14.4	3.6	0.5	270	1.24	0.56	82.2	12.2	5.2	0.4	*ns*
**Administrative work**	243	1.69	0.91	56.4	24.3	13.6	5.8	334	1.81	0.94	48.5	28.7	16.2	6.6	*ns*
**Basic hygiene routines**	262	1.53	0.85	66.4	18.7	10.7	4.2	347	1.35	0.65	73.8	18.4	6.9	0.9	0.005**
**Did not give delegation**	103	1.26	0.58	79.6	15.5	3.9	1.0	121	1.26	0.54	79.3	15.7	5.0	0.0	*ns*
**Did not have delegation**	231	1.09	0.38	93.5	4.3	1.7	0.4	318	1.07	0.33	94.7	3.8	1.3	0.3	*ns*

^†^range 1 “never” to 4 “often”, lower mean indicates less missed nursing care.^‡^ Independent-samples t-test *significance ≤ 0.05, **significance ≤ 0.01

In nursing homes, seldom missed care activities were reported in a range from 3.8% to 37.1%, sometimes missed nursing care ranging from 1.3% to 36.2%, and often missed nursing care ranging from 0.0% to 22.8%. Fifteen out of 35 care activities were missed half of the time to some extent (seldom, sometimes, often). In nursing homes, the most frequently missed care activities were: ‘scheduled group activity’ (22.8%), and ‘scheduled single activity with care recipient’ (19.7%). Ten care activities were reported as never missed in 70% or more of the time. The lowest frequency missed activities were: ‘doing a nursing task when not having a delegation’ (94.7%), and not ‘acting if abuse occurred’ (89.0%), see Table 2 and 3.

Statistically significant differences (*p* ≤ 0.05) were identified in type of missed nursing care between home care and nursing homes, (15 out of 35 items), whereof 11 of these care activities were more often missed in nursing homes than in home care. Five care activities had a statistical significance ≤ 0.01; ‘monitoring care recipient as care worker felt necessary’, ‘monitoring of confused/cognitively impaired care recipients and use of restraints/sedatives’, ‘scheduled single activity with a care recipients’, ‘scheduled group activity with several care recipients’, and ‘assistance while food still hot ‘, were all more often missed in nursing homes. The four nursing tasks more often missed in home care were: ‘studying care plans at the beginning of shift, ‘set up or update care plans’, ‘documentation of care’, and ‘basic hygiene routines’, see Table 2 and 3.

### Reasons for missed nursing care

The results from the open-ended question apply to nurses working in both home care and nursing homes, and it showed consistency for the two settings. Four categories summarize the reasons for missed nursing care: ‘Lack of preparedness for unexpected situations’, ‘Obstacles in a deficient work environment’, ‘Unsatisfactory planning in the organisation’, and ‘Shortcomings related to the individual’.

#### Lack of preparedness for unexpected situations

Participating nurses expressed that if something unforeseen happened or if a care recipient did not want to receive help, there was no margin for the task to take a little longer. There was no time scheduled for unexpected alarms, so it became impossible to carry out all required care. The nurses were forced to prioritize which tasks to do, as there were no opportunity to catch up with everything.

Every day, unforeseen things happen that steal time from the care recipients.

… there is no extra time.

#### Obstacles in a deficient work environment

The nurses stated that missed nursing care could occur when there were deficiencies in communication, such as bad information transfers, language difficulties, or misunderstandings. The nurses had to cover up for co-workers who lacked experience and/or knowledge, because they were new at the workplace, uneducated, or did not have delegation to do all nursing care, which made it hard to complete all required tasks due to time restraints. It was considered time consuming to check that everyone on the team had the same, correct information. The documentation system was too complicated, and was divided for the different professions. Starting and logging into computers was seen as time consuming, and the number of computers was insufficient, so sometimes the documentation was not done.

Regarding administration, it takes a very long time to get into the computer. Sometimes you do not document what you need becauseit takes too long…

#### Unsatisfactory planning in the organisation

Some reasons for missed nursing care were beyond the nurses’ control. The scheduled staffing was experienced as too low and the workload was considered high with too many tasks to do. Participating nurses thought the schedules were poorly planned, which made the working day stressful. When the allotted time for tasks (the tasks are minute-controlled), was not enough, it was impossible to catch up and, even the travel time between care recipients was too short. The nurses expressed that lack of material, such as a medication box that had not been refilled or lack of computers to document the nursing care on, caused missed nursing care.

I want to give care recipient so much more, but it is not possible with the strict minute schedule we follow in home care. Leads to an unsustainable care situation with the risk of unnecessary adverse events that could have been avoided.

#### Shortcomings related to the individual

The nurses described how they did not felt well, and that made them not to perform as well as they usually would. They also expressed feelings of fatigue and they did forget things that ought to done. Sometimes carelessness was the reason that made nursing care been missed.

… you have so much that you forget to pass on important information.

## Discussion

The aim of the study was to describe prevalence, type, and reasons for missed nursing care in home care and nursing homes, from nurses’ perspective. The results of the study indicate that some nursing care activities were performed while others were missed to varying degrees. Rates of missed nursing care were significantly higher in nursing homes than in home care. A possible reason for this could be the organizational differences between home care and nursing homes. In home care nurses visits older people on a scheduled basis doing predetermined nursing activities and in nursing homes several nurses work together in a unit to care for a group of older people with more complex needs. In nursing homes it might be easier to forget something due acute undertakings or to think that someone else will cover up if there is a lack of time to do all required nursing activities. In a national annual survey, 18% of the older people in home care and 28% of older people in nursing homes thinks that the nurses never or seldom have the time to perform all tasks [28]. This is in line with current results that missed nursing care exists in home care, but is more common in nursing homes.

In the present study, many care activities have a high percent for never been missed, indicating that these are high prioritized. On the contrary, low priority is giving to set up care plans, the most frequently missed activity in home care, and the reason was often due to time constraints. This is in agreement with the results from other studies where documentation has been found to have a high frequency of being missed; Phelan, McCarthy and Adams [29] reported 79% missed, and Norman and Sjetne [30] reported 54%. The present result showed that too few computers was seen as a reason for missed nursing care, which is in line with Ausserhofer, Favez, Simon and Zuniga [31] where a significant association between a sufficient number of computers and less missed nursing care was found.

In the present study, nearly half of the time sponge bath/partial sponge bath/skin care was missed to some extent. Another study showed the most common care activity to be missed were assistance with body cleaning [32]. Social activities were an activity often missed in nursing homes. Similar findings where nurse assistants spent almost no time socialising with the older people, though they prioritized most of their time in nursing homes to help residents with personal care [33]. When there is not enough time, priority is given to fundamental care and social care is omitted [15]. The nurses in both home care and nursing homes expressed that sometimes prioritizations must be done, and it was seen as an ethical challenge [34]. Moral distress increased among nurses when quality of life was reduced for older people with dementia due to too few activities. Even having to rush the care because there is not enough time gives a moral distress to the nurses [35].

The results show perceptions of an organisation that is not prepared for unforeseen situations, the organisation is too slim-lined with a too high workload. Organisational factors, including those related to financial austerity and leadership, come with consequences to missed nursing care [36]. When there is not enough time the nurses are forced to prioritize what care to give. This causes dilemmas in how to prioritization due to high workloads, inadequate staffing levels, unexpected events, and conflicting demands [15]. Regardless of the reason, this should be an important focus for managers so they can work on solutions for improvement, so no missed nursing care occurs. When studying reported adverse events in municipal health care circumstances that led to missed nursing care were: insufficient clinical assessments and documentation, not carrying out activities for daily living (e.g. help with hygiene), which in turn led to adverse events [37]. It is known that missed nursing care can lead to serious consequences for older people [36], for patient safety [38], quality of care, and patient satisfaction [39].

### Limitations

There are some limitations to the study. The questionnaire was distributed in different ways, according to how the addresses to e-mails or mail-boxes could be provided, and the way to distribute all have their advantage and disadvantage. For example, 43.5% had the questionnaire distributed by e-mail, and 2.7% were given a hard-copy of the questionnaire on staff meetings. It is a well-known problem that web based surveys can be blocked as spam [40], and never reached the participants. Despite this, no patterns can be seen in response rates according to the way the questionnaire was distributed. The questionnaire is based on self-reported answers, which mean that the answer, deliberately or not, can have bias for social desirability [41]. Perhaps some participants found it controlling or conscientiously difficult to answer questions about what they did not do, but should have done, even if the survey were anonymous. This means that there is a risk of underreporting when studying the phenomenon missed nursing care [9, 42, 43]. Recall bias is a known error [41, 44], it was handled in this study by asking the participants to recall their last seven days. The findings are not different from earlier studies in that the phenomenon missed nursing care exists, even if a generalisation cannot be made yet.

## Conclusions

Nurses’ intentions are to perform all required nursing care activities, but there are care activities that not are performed, for older people in municipal health care. The stated reasons were lack of preparedness for unexpected situations, obstacles in a deficient work environment, unsatisfactory planning in the organisation and/or shortcomings related to the individual. Missed nursing care can lead to adverse events and affect patient safety. Continuously measuring the occurrence of missed nursing care, will make the organisations aware of the phenomenon and give the possibility to prevent it. It also provides an opportunity to take it into consideration when decisions are made at the organisational level, from the head of administration of health care to the managers on respective unit. This should enable improvements to be made and implementation of work procedures that ensure a high patient safety and qualitative of care.

## References

[1] World Health Organization: Ageing and health [https://www.who.int/news-room/fact-sheets/detail/ageing-and-health]

[2] World Health Organisation Europe: Health workforce [https://www.euro.who.int/en/health-topics/Health-systems/health-workforce/health-workforce]

[3] Kalánková D, Žiaková K, Kurucová R (2019). Approaches to understanding the phenomenon of missed/rationed/unfinished care – a literature review. Cent Eur J Nurs Midwif.

[4] Ogletree AM, Mangrum R, Harris Y, Gifford DR, Barry R, Bergofsky L, Perfetto D (2020). Omissions of care in nursing home settings: A narrative review. J Am Med Dir Assoc.

[5] Bragadóttir H, Kalisch BJ, Tryggvadóttir GB (2017). Correlates and predictors of missed nursing care in hospitals. J Clin Nurs.

[6] Kalisch BJ, Landstrom GL, Hinshaw AS (2009). Missed nursing care: A concept analysis. J Adv Nurs.

[7] Zúñiga F, Ausserhofer D, Hamers JPH, Engberg S, Simon M, Schwendimann R (2015). Are staffing, work environment, work stressors, and rationing of care related to care workers' perception of quality of care? A cross-sectional study. J Am Med Dir Assoc.

[8] Ausserhofer D, Zander B, Busse R, Schubert M, De Geest S, Rafferty AM, Ball J, Scott A, Kinnunen J, Heinen M (2014). Prevalence, patterns and predictors of nursing care left undone in european hospitals: Results from the multicountry cross-sectional RN4CAST study. BMJ Qual Saf.

[9] Nelson ST, Flynn L (2015). Relationship between missed care and urinary tract infections in nursing homes. Geriatr Nurs.

[10] Schubert M, De Geest S, Clarke SP, Aiken LH (2012). Associations between rationing of nursing care and inpatient mortality in swiss hospitals. Int J Qual Health Care.

[11] Henderson J, Willis E, Xiao L, Blackman I (2017). Missed care in residential aged care in australia: An exploratory study. Collegian.

[12] Blackman I, Henderson J, Willis E, Weger K (2020). Causal links associated with missed residential aged care. J Nurs Manag.

[13] Campagna S, Basso I, Vercelli E, Ranfone M, Dal Molin A, Dimonte V, Di Giulio P: Missed nursing care in a sample of high-dependency italian nursing home residents: Description of nursing care in action. J Patient Saf*.* 2021;17(8):1840–5. 10.1097/PTS.0000000000000643 32168274

[14] Blackman I, Papastavrou E, Vryonides S, Palese A, Henderson J, Willis E (2018). Predicting variations to missed nursing care: A three-nation comparison. J Nurs Manag.

[15] Ludlow K, Churruca K, Ellis LA, Mumford V, Braithwaite J (2021). Decisions and dilemmas: The context of prioritization dilemmas and influences on staff members' prioritization decisions in residential aged care. Qual Health Res.

[16] National Board of Health and Welfare. Care and nursing for older people - Status report. [Vård och omsorg om äldre - lägesrapport] 2020 2020.

[17] SOU 2017:21: read me! National quality plan for care and nursing for the elderly. Report of the inquiry into a national quality plan for elderly care [Läs mig! Nationell kvalitetsplan för vård och omsorg om äldre personer. Betänkande av utredningen om nationell kvalitetsplan för äldreomsorgen]. Stockholm: Wolters Kluwer.

[18] Swedish Association of Local Authorities and Regions: Kommuner och regioner [municipalities and regions] [https://skr.se/skr/tjanster/kommunerochregioner.431.html]

[19] SFS 2017:30: Health and medical services act. Stockholm: Ministry of Health and Social Affairs.

[20] SFS 2001:453: Social services act. Stockholm: Ministry of Health and Social Affairs.

[21] National Board of Health and Welfare: your right to care and nursing. A guide for the elderly [Din rätt till vård och omsorg. En vägvisare för äldre ]. Stockholm; 2016.

[22] SOSFS 1997:14: delegation of tasks in health care and dentistry [Delegering av arbetsuppgifter inom hälso- och sjukvård och tandvård]. Stockholm: National Board of Health and Welfare.

[23] Zúñiga F, Schubert M, Hamers JPH, Simon M, Schwendimann R, Engberg S, Ausserhofer D (2016). Evidence on the validity and reliability of the german, french and italian nursing home version of the basel extent of rationing of nursing care instrument. J Adv Nurs.

[24] Field AP (2018). Discovering statistics using IBM SPSS statistics.

[25] Graneheim UH, Lindgren B, Lundman B (2017). Methodological challenges in qualitative content analysis: A discussion paper. Nurse Educ Today.

[26] Graneheim UH, Lundman B (2004). Qualitative content analysis in nursing research: Concepts, procedures and measures to achieve trustworthiness. Nurse Educ Today.

[27] World Medical Association: Declaration of Helsinki – ethical principles for medical research involving human subjects [https://www.wma.net/policies-post/wma-declaration-of-helsinki-ethical-principles-for-medical-research-involving-human-subjects/]

[28] National Board of Health and Welfare: what do the elderly think about elderly care? 2019 [Vad tycker de äldre om äldreomsorgen?]; 2019.

[29] Phelan A, McCarthy S, Adams E (2018). Examining missed care in community nursing: A cross section survey design. J Adv Nurs.

[30] Norman RM, Sjetne IS (2019). Adaptation, modification, and psychometric assessment of a norwegian version of the basel extent of rationing of nursing care for nursing homes instrument (bernca-nh). BMC Health Serv Res.

[31] Ausserhofer D, Favez L, Simon M, Zuniga F (2021). Electronic health record use in swiss nursing homes and its association with implicit rationing of nursing care documentation: Multicenter cross-sectional survey study. JMIR Med Inform.

[32] Tou YH, Liu MF, Chen SR, Lee PH, Kuo LM, Lin PC (2020). Investigating missed care by nursing aides in taiwanese long-term care facilities. J Nurs Manag.

[33] Mallidou AA, Cummings GG, Schalm C, Estabrooks CA (2013). Health care aides use of time in a residential long-term care unit: A time and motion study. Int J Nurs Stud.

[34] Munkeby H, Moe A, Bratberg G, Devik SA (2021). 'Ethics between the lines' – nurses' experiences of ethical challenges in long-term care. Global Qualitative Nursing Research.

[35] Pijl-Zieber EM, Awosoga O, Spenceley S, Hagen B, Hall B, Lapins J (2018). Caring in the wake of the rising tide: Moral distress in residential nursing care of people living with dementia. Dementia.

[36] Sworn K, Booth A (2020). A systematic review of the impact of ‘missed care’ in primary, community and nursing home settings. J Nurs Manag.

[37] Andersson Å, Frank C, Willman AML, Sandman PO, Hansebo G (2018). Factors contributing to serious adverse events in nursing homes. J Clin Nurs.

[38] Jangland E, Teodorsson T, Molander K, Muntlin Athlin Å (2018). Inadequate environment, resources and values lead to missed nursing care: A focused ethnographic study on the surgical ward using the fundamentals of care framework. J Clin Nurs.

[39] Zhao Y, Ma D, Wan Z, Sun D, Li H, Sun J (2020). Associations between work environment and implicit rationing of nursing care: A systematic review. J Nurs Manag.

[40] Bowling MJ, Rimer BK, Lyons EJ, Golin CE, Frydman G, Ribisl KM (2006). Methodologic challenges of e-health research. Eval Program Plann.

[41] Streiner DL, Norman GR, Cairney J (2015). Health measurement scales : A practical guide to their development and use.

[42] Zúñiga F, Ausserhofer D, Hamers JPH, Engberg S, Simon M, Schwendimann R (2015). The relationship of staffing and work environment with implicit rationing of nursing care in swiss nursing homes - a cross-sectional study. Int J Nurs Stud.

[43] Senek M, Ryan T, King R, Wood E, Tod A, Robertson S, Sworn K (2020). Nursing care left undone in community settings: Results from a uk cross-sectional survey. J Nurs Manag.

[44] Althubaiti A (2016). Information bias in health research: Definition, pitfalls, and adjustment methods. J Multidiscip Healthc.

